# A meta-analysis of clinical benefit rates for fulvestrant 500 mg vs. alternative endocrine therapies for hormone receptor-positive advanced breast cancer

**DOI:** 10.1007/s12282-019-00973-4

**Published:** 2019-05-11

**Authors:** John F. R. Robertson, Zefei Jiang, Angelo Di Leo, Shinji Ohno, Kathleen I. Pritchard, Matthew Ellis, Ian Bradbury, Christine Campbell

**Affiliations:** 1 Division of Breast Surgery, The University of Nottingham, Nottingham City Hospital, Hucknall Road, Nottingham, NG5 1PB UK; 2 Department of Breast Cancer, Affiliated Hospital of Academy of Military Medical Sciences, No. 8 Dongda Street, Fengtai District, Beijing, 100071 China; 3 “Sandro Pitigliani” Medical Oncology Department, Hospital of Prato, Azienda USL Toscana Centro, Piazza Dell’ospedale 2, 59100 Prato, Italy; 4 Breast Oncology Center, Cancer Institute Hospital, 3-8-31 Ariake, Koto-ku, Tokyo, 135-8550 Japan; 5 Sunnybrook Odette Cancer Centre and the University of Toronto, 2075 Bayview Ave, Toronto, ON M4N 3M5 Canada; 6 Lester and Sue Smith Breast Center, Baylor College of Medicine, 6620 S Main St #1350, Houston, TX 77030 USA; 7 Frontier Science, Grampian View, Kincraig, Kingussie, PH21 1NA UK

**Keywords:** Advanced breast cancer, Clinical benefit rate, Fulvestrant, Hormone receptor-positive, Meta-analysis

## Abstract

**Background:**

Fulvestrant, a selective estrogen receptor degrader, is approved for first- and second-line treatment of postmenopausal women with hormone receptor-positive advanced breast cancer (ABC).

**Methods:**

Meta-analysis of randomized controlled trials (RCTs) evaluating fulvestrant 500 mg in postmenopausal hormone receptor-positive ABC, to evaluate differences in clinical benefit rate (CBR; proportion of patients experiencing best overall response of complete response, partial response, or stable disease for ≥ 24 weeks) between fulvestrant 500 mg and comparator endocrine therapies. Odds ratios (OR) and 95% confidence intervals (CI) for CBR were calculated; fixed effects (FE) models were constructed (first- and second-line data, alone and combined).

**Results:**

Six RCTs were included. Four studies evaluated fulvestrant 500 mg vs. fulvestrant 250 mg; two evaluated fulvestrant 500 mg vs. anastrozole 1 mg. In total, 1054 and 534 patients were included (first- and second-line treatment, respectively). Analysis of OR and 95% CI of CBR by therapy line favored fulvestrant 500 mg vs. comparator therapy. Assessing all results combined in the FE model indicated significant improvement in CBR with fulvestrant 500 mg vs. comparator treatments (OR 1.33; 95% CI 1.13–1.57; *p *= 0.001). Restricting the FE model to therapy line demonstrated significant improvement in CBR vs. comparator treatments (OR 1.33; 95% CI 1.02–1.73; *p *= 0.035) for first-line, and a trend to improvement vs. comparator treatments (OR 1.27; 95% CI 0.90–1.79; *p *= 0.174) for second-line.

**Conclusions:**

In postmenopausal patients with hormone receptor-positive ABC, fulvestrant 500 mg first-line was associated with significantly greater CBR (more patients benefiting from treatment) vs. comparator endocrine therapy.

## Introduction

The majority of patients diagnosed with breast cancer have hormone receptor-positive (HR+) disease [1]. Endocrine therapy (ET) is recommended as first- and second-line treatment for the majority of postmenopausal women with HR+ advanced breast cancer (ABC), based on empirical evidence showing the clinical effectiveness of these agents [2–4].

Fulvestrant is a selective estrogen receptor (ER) degrader that binds to, and blocks, the ER, while increasing ER degradation [5]. Fulvestrant 500 mg is approved for the treatment of postmenopausal women with ER-positive ABC and disease progression following failure on prior antiestrogen therapy [6, 7].

In 2017, fulvestrant was approved by the European Medicines Agency, the US Food and Drug Administration, Japan, and Russia, for the first-line treatment of postmenopausal patients with HR+ locally advanced or metastatic breast cancer who have not received prior ET [6–8]. Furthermore, for second-line patients who have disease progression after prior ET, fulvestrant is now approved in the US and Europe in combination with the cyclin-dependent kinase 4/6 (CDK4/6) inhibitor palbociclib, and in the US with the CDK4/6 inhibitor abemaciclib [6, 7].

The 2017 approval of fulvestrant as monotherapy in the first-line setting was based on the results of the phase III, randomized, double-blind FALCON study in endocrine-naïve patients with ABC, in which progression-free survival (PFS) was significantly longer with fulvestrant 500 mg than anastrozole [9]. Overall survival (OS) data from this trial have not yet been reported, as data are immature. Moreover, in the randomized, open-label, phase II FIRST study in postmenopausal women with ABC, fulvestrant 500 mg improved time to progression (TTP) and OS vs. anastrozole as first-line treatment [10–12]. Similarly, in the second-line setting, PFS and OS advantages were reported with fulvestrant 500 mg compared with fulvestrant 250 mg in the phase III, randomized, double-blind CONFIRM study in patients with ABC who had progressed on prior ET [13, 14].

Historically, OS has been considered a key endpoint in clinical trials. However, as a measure of tumor response, OS can be confounded by post-trial therapies and deaths unrelated to cancer [15]. PFS and TTP measurements, while objective (particularly when performed in a double-blinded trial, or with external review), reflect the time taken for the disease to become resistant to a given therapy, and for tumor growth to advance. However, the durations of PFS and TTP do not provide a direct indication of whether objective measures of tumor response are improved during this period. Although OS and TTP/PFS are measures commonly used to determine efficacy in clinical trials, some studies in patients with ABC have used clinical benefit rate (CBR) as a clinical endpoint. For instance, the FIRST study of first-line fulvestrant vs. anastrozole was designed with CBR as the primary endpoint [10]. CBR reflects the proportion of patients who experience a treatment benefit with an ET—either by tumor remission or prolonged periods of disease stability—and provides an estimate of the number of patients experiencing a positive tumor response to anticancer therapy. Questions remain regarding how closely this outcome is correlated to OS. However, in patients with ABC, it is known that those with stable disease have similar survival curves to those with an objective response [16–18].

To date, few clinical trials have reported a significant improvement in CBR with one ET compared with another for the treatment of ABC [19, 20]. Therefore, we performed a meta-analysis of data from randomized controlled trials (RCTs) to determine if there is a difference in CBR between fulvestrant 500 mg and other ETs.

## Materials and methods

### Study design and data extraction

We reviewed MEDLINE for English language articles published before June 2016 (date of analysis) that reported CBRs from an RCT assessing fulvestrant 500 mg vs. a comparator monotherapy for the treatment of HR+ ABC in postmenopausal women. Fulvestrant at the 250 mg dose was considered a comparator ET based on previous results that have shown fulvestrant 250 mg to be at least as effective as anastrozole in terms of TTP, objective response, and duration of response in the second-line treatment of patients with ABC [21].

From the literature review, six RCTs evaluating fulvestrant 500 mg for the treatment of HR+ ABC in postmenopausal women were identified and included in the meta-analysis (Table 1).

**Table 1:** Study design details and baseline patient treatment characteristics for included trials reporting on postmenopausal women with HR+ locally advanced or metastatic breast cancer

FALCON [9]	FIRST [10–12]	CONFIRM [13, 14]	FINDER1 [22]	FINDER2 [23]	China CONFIRM [20]
Study design					
Phase III, randomized, double-blind, multicenter study in postmenopausal women with ER+ and/or PgR+ locally advanced or metastatic breast cancer (NCT01602380)	Phase II, randomized, open-label, multicenter, parallel-group study in postmenopausal women with ER+ and/or PgR+ ABC (NCT00274469)	Phase III, randomized, double-blind, multicenter, parallel-group study in postmenopausal women with ER+ ABC (NCT00099437)	Phase II, randomized, double-blind, parallel-group study, conducted in Japan, in postmenopausal women with ER+ ABC (NCT00305448)	Phase II, randomized, double-blind, parallel-group, international study in postmenopausal women with ER+ ABC (NCT00313170)	Phase III, randomized, double-blind study, conducted in China in postmenopausal women with ER+ ABC (NCT01300351)
Treatment arms (number of randomized patients)					
Fulvestrant 500 mg (*n *= 230) vs. anastrozole 1 mg (*n *= 232)	Fulvestrant 500 mg (*n *= 102) vs. anastrozole 1 mg (*n *= 103)	Fulvestrant 500 mg (*n *= 362) vs. fulvestrant 250 mg (*n *= 374)	Fulvestrant 500 mg (*n *= 47) vs. fulvestrant 250 mg (*n *= 45)	Fulvestrant 500 mg (*n *= 46) vs. fulvestrant 250 mg (*n *= 47)	Fulvestrant 500 mg (*n *= 111) vs. fulvestrant 250 mg (*n *= 110)
Treatment line					
First-line Patients were not permitted to have received prior hormone therapy for breast cancer	First-line Prior ET for advanced disease was not permitted Patients could have received adjuvant ET for early disease, if completed > 12 months before randomization	First- and second-line Patients may have experienced relapse on adjuvant ET or < 1 year from completion of adjuvant ET	Second-line Patients may have relapsed during, or ≤ 12 months after, adjuvant ET, or may be progressing on ET started ≥ 12 months after prior adjuvant ET or for de novo advanced disease	Second-line Patients may have relapsed during or ≤ 12 months after adjuvant ET, or may be progressing on ET started ≥ 12 months after prior adjuvant ET or for de novo advanced disease	First- and second-line Patients may have relapsed during or ≤ 12 months after adjuvant ET, or may be progressing on ET started ≥ 12 months after prior adjuvant ET or for de novo advanced disease
Median age (range), years					
64 (38–87) vs. 62 (36–90)	66 (40–89) vs. 68 (48–87)	61 (NR) vs. 61 (NR)	61 (45–83) vs. 61 (50–77)	67 (49–85) vs. 63 (42–88)	55 (26–80) vs. 55 (31–76)
Visceral involvement, %					
59 vs. 51	47.1 vs. 56.3	66 vs. 62	57.4 vs. 57.8	80.4 vs. 72.3	NR
Prior ET, %					
1 vs. < 1	28.4 vs. 22.3	100 vs. 100	100 vs. 100 Anastrozole: 57.4 vs. 57.8 Tamoxifen: 48.9 vs. 42.2 Exemestane: 17.0 vs. 20.0	Anastrozole: 37.0 vs. 38.3 Tamoxifen: 58.7 vs. 59.6 Exemestane: 34.8 vs. 23.4	Adjuvant: 97.3 vs. 93.6 Advanced disease: 31.5 vs. 27.3
Prior chemotherapy (advanced disease), %					
16 vs. 19	0 vs. 0	22.4 vs. 18.4 [29]	70.2 vs. 55.6	56.5 vs. 59.6	22.5 vs. 18.2

*ABC* advanced breast cancer, *ER+ * estrogen receptor-positive, *ET* endocrine therapy, *HR+ * hormone receptor-positive, *NR* not reported, *PgR+ * progesterone receptor-positive

These were the Fulvestrant and AnastrozoLe COmpared in hormonal therapy-Naïve ABC study (FALCON, NCT01602380), a phase III, randomized, double-blind, double-dummy, international trial comparing fulvestrant 500 mg (days 0, 14, 28, then every 28 days thereafter) with anastrozole 1 mg once daily (QD) in the first-line setting [9]; the Fulvestrant fIRst-line Study comparing endocrine Treatments (FIRST, NCT00274469), a phase II international trial comparing fulvestrant 500 mg (days 0, 14, 28, then every 28 days thereafter) with anastrozole 1 mg QD in the first-line setting [10–12]; COmparisoN of Faslodex In Recurrent or Metastatic breast cancer (CONFIRM, NCT00099437), a phase III international trial comparing fulvestrant 500 mg (days 0, 14, 28, then every 28 days thereafter) with fulvestrant 250 mg (days 0, 14, 28, then every 28 days thereafter) in the first- and second-line settings [13, 14]; Faslodex INvestigation of Dose evaluation in Estrogen Receptor-positive ABC (FINDER1, NCT00305448), a phase II trial, conducted in Japan, that compared fulvestrant 500 mg (days 0, 14, 28, then every 28 days thereafter) with fulvestrant 250 mg (250 mg fulvestrant on days 0, 28, then every 28 days thereafter, with placebo injections given on day 14) and fulvestrant loading dose (initial dose of 500 mg fulvestrant at day 0 and 250 mg fulvestrant and placebo on days 14, 28, then every 28 days thereafter) in the second-line setting [22]; FINDER2 (NCT00313170), a phase II trial, conducted in Canada and Europe, that compared fulvestrant 500 mg (days 0, 14, 28, then every 28 days thereafter) with fulvestrant 250 mg (250 mg fulvestrant on days 0 and 28 and every 28 days thereafter, with placebo injections given on day 14) and fulvestrant loading dose (initial dose of 500 mg fulvestrant at day 0 and 250 mg fulvestrant and placebo on days 14, 28, then every 28 days thereafter) in the second-line setting [23]; and China CONFIRM (NCT01300351), a phase III trial, conducted in China, that compared fulvestrant 500 mg (days 0, 14, 28, then every 28 days thereafter) with fulvestrant 250 mg (days 1, 28, and every 28 days thereafter, with placebo injections given on day 14) in the first- and second-line settings [20].

One additional study—the phase II, randomized, open-label Neoadjuvant Endocrine Therapy for Women with Estrogen-Sensitive Tumors (NEWEST, NCT00093002) study [24] of fulvestrant 500 mg vs. fulvestrant 250 mg—was excluded from the meta-analysis, as the study duration was only 16 weeks and tumor responses were not assessed using Response Evaluation Criteria In Solid Tumors (RECIST) v1.1 criteria.

The CBR for each therapy arm in the studies was calculated as the proportion of all randomized patients experiencing a best overall response of complete response or partial response, or a best objective response of stable disease for ≥ 24 weeks. Response subcategories were defined according to RECIST v1.1 criteria [25].

Complete response was defined as the disappearance of all target lesions and non-target lesions, with no new lesions observed, whereas partial response was considered a  > 30% decrease in the sum of diameters of target lesions (compared with baseline), with no progression of non-target lesions and no new lesions. Patients with stable disease had neither sufficient shrinkage of target lesions (i.e., 30% shrinkage) to qualify as a partial response, nor sufficient growth (i.e., 20% increase in the sum of longest diameter of the target lesions, compared with previous smallest sum) to qualify as progression, with no evidence of progression of non-target lesions and no new lesions.

Patients with non-measurable disease at baseline were classed at follow-up as having experienced disease progression if there was evidence of new lytic bone lesions, new lesions outside of the bone, or unequivocal progression of existing bone lesions.

In all studies, patients provided written informed consent and study approval was obtained from independent ethics committees at every study centre. Each study was undertaken in accordance with local legal and regulatory requirements and the general principles of the International Ethical Guidelines for Biomedical Research Involving Human Subjects, the International Conference on Harmonisation guidelines on Good Clinical Practice, and the Declaration of Helsinki.

### Statistical analyses

The Peto method was used to calculate odds ratios (OR), 95% confidence intervals (CI), and corresponding *p*-values [26]. The Peto method for pooled ORs is an alternative to the Mantel–Haenszel method [27]. It is more robust to missing data than the Mantel–Haenszel method when effect sizes are small and can only be used when within-study group sizes are similar and effect sizes are not large [26]. In this meta-analysis, the application of either the Mantel–Haenszel method or the Peto method would be inconsequential.

Unadjusted OR for CBR for fulvestrant vs. comparator was used in the FALCON, FIRST, CONFIRM, FINDER1, and FINDER2 studies. Adjusted OR was used in China CONFIRM, owing to the stratified randomization scheme used in that study [20]. Due to the different doses of fulvestrant used, data for the fulvestrant loading dose (initial dose of 500 mg fulvestrant at day 0, and 250 mg fulvestrant and placebo on days 14, 28, then every 28 days thereafter) in the FINDER1 and FINDER2 studies were not included in the analysis. An OR for CBR > 1.0 was considered to favor fulvestrant vs. comparator, an OR of 1.0 indicated no difference between treatments, and an OR < 1.0 was considered to favor the comparator over fulvestrant.

Fixed effects (FE) models were constructed for first- and second-line data, alone and combined. For each model, a Tarone’s test for heterogeneity was used to assess the assumption of constant trial effect [28]. OR for CBR with fulvestrant 500 mg vs. comparator treatments, and corresponding 95% CI, were calculated.

## Results

From the literature review, six eligible studies were identified for inclusion in this meta-analysis. The study designs and baseline prior treatment characteristics of participating patients in each study are summarized in Table 1.

Of the studies included in the meta-analysis, four (two phase II, two phase III) evaluated fulvestrant 500 mg in comparison with fulvestrant 250 mg, and two (one phase II, one phase III) evaluated fulvestrant 500 mg in comparison with anastrozole 1 mg. The results for efficacy outcomes for each treatment arm in each study are shown in Table 2. In studies with evaluable data, the median PFS/TTP with fulvestrant 500 mg ranged from  5.6 to 23.4 months and 6.0 to 7.9 months in the first- and second-line settings, respectively. Objective response rates were 11.2–46.1% and 10.6–15.7% in the first and second lines, respectively.

**Table 2:** Efficacy endpoint results for included trials reporting on postmenopausal women with HR + locally advanced or metastatic breast cancer

FALCON [9]	FIRST [10–12]	CONFIRM [13, 14, 30]			FINDER1 [22]	FINDER2 [23]	China CONFIRM^a^ [20]
Treatment arms (number of randomized patients)							
First-line setting: Fulvestrant 500 mg (*n *= 230) vs. anastrozole 1 mg (*n *= 232)	First-line setting: Fulvestrant 500 mg (*n *= 102) vs. anastrozole (*n *= 103)	Overall: Fulvestrant 500 mg (*n *= 362) vs. fulvestrant 250 mg (*n *= 374)	First-line setting: Fulvestrant 500 mg (*n *= 191) vs. fulvestrant 250 mg (*n *= 196)^b^	Second-line setting: Fulvestrant 500 mg (*n *= 166) vs. fulvestrant 250 mg (*n *= 177)^b^	Second-line setting: Fulvestrant 500 mg (*n *= 47) vs. fulvestrant 250 mg (*n *= 45)	Second-line setting: Fulvestrant 500 mg (*n *= 46) vs. fulvestrant 250 mg (*n *= 47)	Overall: Fulvestrant 500 mg (*n *= 111) vs. fulvestrant 250 mg (*n *= 110)
CBR, %							
78.3 vs. 74.1; OR 1.25; 95% CI 0.82–1.93; *p *=0.305	Primary endpoint: 72.5 vs. 67.0; OR 1.30; 95% CI 0.72–2.38; *p *= 0.386	45.6 vs. 39.6; OR 1.28; 95% CI 0.95–1.71; *p *=0.100	44.0 vs. 35.7; OR 1.41; 95% CI 0.94–2.13; *p *= 0.097^c^	47.4 vs. 43.8; OR 1.15; 95% CI 0.76–1.76; *p *= 0.506^c^	46.8 vs. 42.2	47.8 vs. 31.9	47.7 vs. 32.7; OR 1.37; 95% CI 1.04–1.80; *p *= 0.023
Median PFS/TTP, months							
Primary endpoint: 16.6 vs. 13.8; hazard ratio 0.797; 95% CI 0.637–0.999; *p *= 0.0486	23.4 vs. 13.1; hazard ratio 0.66; 95% CI 0.47–0.92; *p *= 0.01	Primary endpoint: 6.5 vs. 5.5; hazard ratio 0.80; 95% CI 0.68–0.94; *p *= 0.006	5.6 vs. 4.2; hazard ratio 0.80; 95% CI 0.64–1.00; *p *= 0.047	7.9 vs. 6.3; hazard ratio 0.80; 95% CI 0.64–1.02; *p *= 0.068	6.0 vs. 6.0	6.0 vs. 3.1	Primary endpoint: 8.0 vs. 4.0; hazard ratio 0.75; 95% CI 0.54–1.03; *p *= 0.078
ORR, %							
46.1 vs. 44.9	36.0 vs. 35.5	9.1 vs. 10.2	11.2 vs. 14.5	15.7 vs. 14.7	Primary endpoint: 10.6 vs. 11.1	Primary endpoint: 15.2 vs. 8.5	28.1 vs. 16.7

*CBR* clinical benefit rate, *CI* confidence interval, *HR+ * hormone receptor-positive, *NM* not mature, *NR* not reported, *OR* odds ratio, *ORR* objective response rate, *PFS* progression-free survival, *TTP* time to progression

^a^China CONFIRM recruited first- and second-line patients; however, results by line were not available

^b^All efficacy endpoints, except for CBR, were based on the respective patient populations. Six randomized patients were excluded from these analyses; three first-line patients experienced relapse > 12 months after completion of prior hormone therapy, two received fulvestrant treatment in the third-line, and there was insufficient information on prior therapies received for one patient. CBR was calculated based on the respective patient populations in the first-line (fulvestrant 500 mg, *n *= 191; fulvestrant 250 mg, *n *= 196) and second-line settings (fulvestrant 500 mg, *n *= 171; fulvestrant 250 mg, *n *= 178)

^c^First- and second-line CBR data for CONFIRM were calculated for this analysis, and have not been previously published

In total, 1809 patients were included in the meta-analysis of CBR. As line-of-treatment data for China CONFIRM were not available, these patients (*n *= 221) were not included in subsequent analyses according to line of treatment. Therefore, 1054 and 534 patients (1588 in total) were included in the analysis of first- and second-line treatment, respectively.

**Fig. 1:**
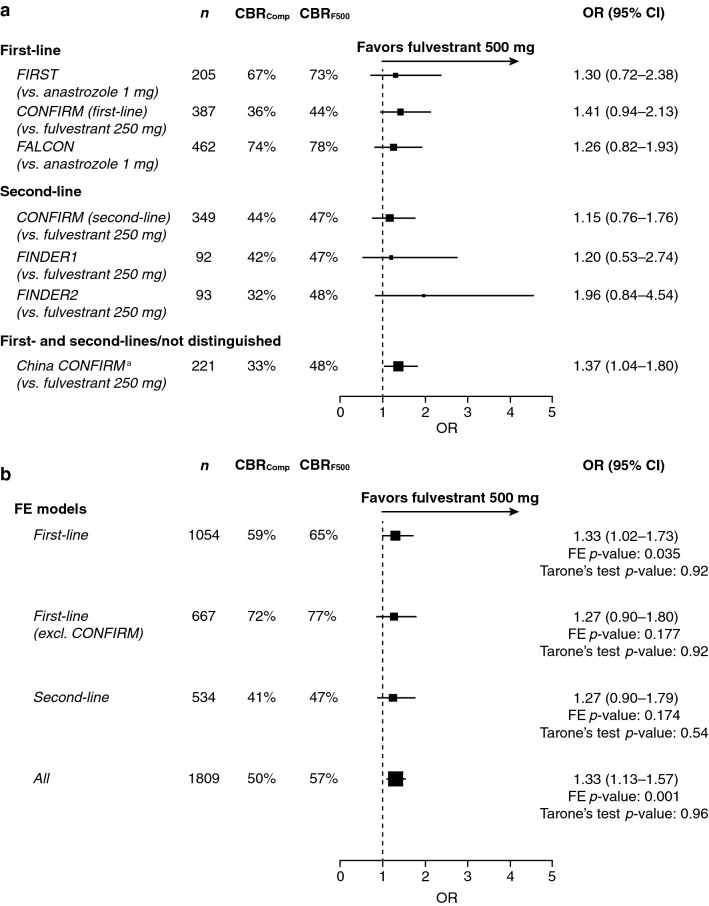
OR and 95% CI for CBR during treatment with fulvestrant 500 mg vs. comparator in **a** individual trials and **b** the FE models. *CBR*_*Comp*_ clinical benefit rate for patients receiving comparator therapy, *CBR*_*F500*_ clinical benefit rate for patients receiving fulvestrant 500 mg therapy, *CI* confidence interval, *excl* excluding, *FE* fixed effects, *OR* odds ratio. ^a^China CONFIRM recruited first- and second-line patients; however, results by line were not available

Analysis of OR and 95% CI of the CBR by line of therapy favored fulvestrant 500 mg vs. comparator therapy during the treatment of HR+ ABC for each trial (Fig. 1a), in both the first- and second-line settings.

When the FE model was used to assess all combined first- and second-line trial results, the OR indicated that fulvestrant 500 mg was associated with a significant improvement in CBR vs. comparator treatments (OR 1.33; 95% CI 1.13–1.57; FE model *p *=0.001; Tarone’s test *p *=0.96; Fig. 1b).

Restricting the FE model to the first-line setting demonstrated a significant improvement in CBR vs. comparator treatments (OR 1.33; 95% CI 1.02–1.73; FE model *p *=0.035; Tarone’s test *p *=0.92). When the FE model was restricted to the second-line setting, the OR indicated that fulvestrant 500 mg was associated with a numeric improvement in CBR vs. comparator treatments (OR 1.27; 95% CI 0.90–1.79; FE model *p *=0.174; Tarone’s test *p *=0.54; Fig. 1b). A sensitivity analysis directly comparing fulvestrant 500 mg with anastrozole in the first-line setting was also performed. This excluded the data from CONFIRM and looked at the FIRST and FALCON trials combined (Fig. 1b). This showed that fulvestrant 500 mg was associated with a numeric improvement in CBR vs. anastrozole (OR 1.27; 95% CI 0.90–1.80; FE model *p* = 0.177; Tarone’s test *p* = 0.92; Fig. 1b).

For all models, the Tarone’s test for heterogeneity was not significant (*p *=0.92, 0.54, and 0.96 for first-line, second-line, and all patients, respectively).

## Discussion

In this meta-analysis, we investigated the CBR for fulvestrant 500 mg vs. alternative ETs for the treatment of postmenopausal women with HR+ ABC. From our literature review, we identified six eligible studies reporting on data comparing fulvestrant 500 mg with other ETs. Across all studies evaluated, the ORs for CBR were favorable for fulvestrant 500 mg vs. anastrozole or fulvestrant 250 mg.

From the FE model, the findings suggest that fulvestrant 500 mg is associated with a significant improvement in CBR of ~ 33% vs. comparator ETs (i.e., more tumors enter remission or prolonged stability with fulvestrant 500 mg). Further analysis of CBR by line of therapy demonstrated a significant improvement in CBR of ~ 33% in the first-line setting, and a trend to improvement of ~ 27% in the second-line setting. Fulvestrant 250 mg has been shown to be equivalent to an aromatase inhibitor (AI) in the second-line setting [21]. In the CONFIRM trial, we used fulvestrant 250 mg as a surrogate for an AI. We acknowledge that there has never been a direct comparison of fulvestrant 250 mg dose vs. an AI in the first-line setting, as there has been in the second-line setting. We therefore carried out a sensitivity analysis that omitted the CONFIRM first-line data. This showed a very similar OR (1.27) to that observed in the second-line setting (1.27) and overall for all of the studies (1.33). We therefore feel that this supports our initial approach of combining FIRST, FALCON, and CONFIRM first-line patients.

This observation was relatively consistent across trials. However, based on coverage of 95% CI, individual studies generally reported non-inferiority, rather than superiority, of CBR with fulvestrant 500 mg vs. comparator therapy (anastrozole in the FIRST study, and fulvestrant 250 mg in all other studies).

These results provide important context for PFS/TTP and OS data, and suggest that—in addition to delaying disease progression—the odds of experiencing a positive tumor response or disease control are significantly increased for patients receiving fulvestrant 500 mg. Of note, the duration of clinical benefit was longer with fulvestrant 500 mg treatment compared with comparator treatment in all studies [9–14, 20, 22, 23]; the improvement in PFS with fulvestrant 500 mg in these studies may therefore reflect both its longer duration of clinical benefit (i.e., delay in developing acquired resistance) and the fact that more patients are placed into remission (i.e., clinical benefit). As such, CBR provides clinicians with an objective outcome measure to determine the proportion of patients with a tumor response to treatment, a factor that may not be clearly identified by survival measures such as PFS/TTP and OS.

In addition to patients with a complete or partial response to treatment, CBR also includes patients with stable disease (i.e., no disease progression for at least 24 weeks). Although these patients do not experience disease shrinkage, previous work has demonstrated that subsequent survival among patients experiencing stable disease for at least 6 months does not differ significantly from that of patients achieving partial response [16–18].

One potential limitation of the analysis could be the smaller number of patients who received fulvestrant 500 mg as second- compared with first-line therapy (534 and 1054 patients, respectively). This may account for why the FE model analysis of CBR with fulvestrant 500 mg vs. comparators in the second-line setting was not statistically significant. Furthermore, we acknowledge that there is no direct comparison of fulvestrant 500 mg vs. anastrozole in patients treated in the first-line for ABC who have received prior adjuvant AIs. However, we would view this as strengthening the conclusions that fulvestrant 500 mg places more patients into clinical benefit. If there had been prior exposure to an adjuvant AI, then it could have been argued that a comparison of fulvestrant 500 mg vs. an AI in the first-line ABC setting was biased against the AI. This is something that cannot be leveled at the comparison reported in the meta-analysis.

A relevant consideration when determining the effectiveness of any treatment is how well the treatment is tolerated, and this analysis does not provide an indication of the comparative tolerability profiles of each treatment, although individual studies reported similar tolerability profiles between fulvestrant 500 mg and the comparator arms of these trials (i.e., fulvestrant 250 mg or anastrozole 1 mg) [9–14, 20, 22, 23].

In conclusion, the findings from this meta-analysis suggest that, in postmenopausal patients with HR+ ABC, fulvestrant 500 mg is associated with significant improvements in CBR compared with comparator ET when evaluating combined first- and second-line trial results, i.e., significantly more patients are placed in remission by this treatment than comparators. Restricting our analysis to line of therapy demonstrated that fulvestrant 500 mg was associated with a significant improvement in CBR in first-line treatment. In the second-line setting, there was a trend to improvement; while this did not reach statistical significance, the test for heterogeneity indicates that there was no evidence that the treatment effects were not consistent across the first- and second-line studies. Together, these findings add to the evidence base supporting the effectiveness of fulvestrant 500 mg in postmenopausal women with HR+ ABC.
